# Conference Didactic Planning and Structure: An Evidence-based Guide to Best Practices from the Council of Emergency Medicine Residency Directors

**DOI:** 10.5811/westjem.2020.5.46762

**Published:** 2020-07-03

**Authors:** D. Brian Wood, Jaime Jordan, Rob Cooney, Katja Goldflam, Leah Bright, Michael Gottlieb

**Affiliations:** * St. Joseph’s Medical Center, Department of Emergency Medicine, Stockton, California; † Ronald Reagan UCLA Medical Center, David Geffen School of Medicine, Department of Emergency Medicine, Los Angeles, California; ‡ Geisinger Commonwealth School of Medicine, Department of Emergency Medicine, Scranton, Pennsylvania; § Yale University, Department of Emergency Medicine, New Haven, Connecticut; ¶ Johns Hopkins University, Department of Emergency Medicine, Baltimore, Maryland; || Rush University Medical Center, Department of Emergency Medicine, Chicago, Illinois

## Abstract

Emergency medicine residency programs around the country develop didactic conferences to prepare residents for board exams and independent practice. To our knowledge, there is not currently an evidence-based set of guidelines for programs to follow to ensure maximal benefit of didactics for learners. This paper offers expert guidelines for didactic instruction from members of the Council of Emergency Medicine Residency Directors Best Practices Subcommittee, based on best available evidence. Programs can use these recommendations to further optimize their resident conference structure and content. Recommendations in this manuscript include best practices in formatting didactics, selection of facilitators and instructors, and duration of individual sessions. Authors also recommend following the Model of Clinical Practice of Emergency Medicine when developing content, while incorporating sessions dedicated to morbidity and mortality, research methodology, journal article review, administration, wellness, and professionalism.

## BACKGROUND

Graduate medical educators are responsible for training well-rounded physicians who are prepared to practice their specialty independently following graduation. A significant component of their education comes from “regularly scheduled didactic sessions” as prescribed by the Accreditation Council for Graduate Medical Education (ACGME) Common Program Requirements.^1^ Specialty-specific ACGME requirements provide further recommendations regarding the amount of dedicated didactic time that must be provided and general themes that must be covered such as journal review, morbidity and mortality (M&M) conference, and research seminars.^2^

Beyond these general requirements, programs have the flexibility to develop their conference structure and content however they choose. This has led to much variation in conference structure, content, and how specific conferences such as M&M are conducted at each individual program. This variation leads to very different experiences for residents based on the residency they attend and has the potential of producing graduates with uneven exposure to key didactic topics during their training. To our knowledge, no evidence-based guidelines or best practices exist to aid educators in the design or implementation of residency didactic curricula. This article provides an evidence-based summary of the literature and best practice recommendations for didactics as it pertains to conference structure and content with a focus on emergency medicine (EM) residency programs.

## CRITICAL APPRAISAL OF THE LITERATURE

This is the fifth article in a series by the Council of Emergency Medicine Residency Directors (CORD) Best Practices Subcommittee.^3^–^6^ A medical librarian performed a search of Medline, Embase, Web of Science, CINAHL, and ERIC for articles published from inception of each database through February 7, 2019, using keyword combinations of education level (medical, graduate, internship, house staff, PGY, and residency), as well as didactics (or conference or lecture) with differing frequency (daily, weekly, bi-weekly, and monthly). Two authors then screened each of the articles independently for papers addressing the three themes: conference structure; conference topics; and M&M sessions. Given the extent of M&M literature obtained, the authors decided to separate articles dedicated specifically to that topic.

The initial literature search yielded 1,199 articles, which were then categorized and provided to content experts for consideration of inclusion in this review. A total of 101 articles were selected for inclusion in this review. Each best practice statement has a corresponding level of evidence and grade based on the Oxford Centre for Evidence-Based Medicine criteria (Tables 1 and 2).^7^ When there was insufficient supporting data, the authors based recommendations on their experience and consensus opinion. The entire CORD Best Practices Subcommittee reviewed the manuscript after which time it was posted on the CORD website for review by the entire CORD community.

## OVERALL CONFERENCE STRUCTURE

Many factors may influence programmatic decisions regarding timing, frequency, and duration of didactic curricula in addition to the desire to optimize education. These may include regulatory requirements, clinical work schedules, locations of faculty and trainees, personnel (teachers and learners), and space availability. The concentrated blocked weekly didactic format (i.e., a single, dedicated conference half day per week) is highly prevalent in other specialties such as family medicine and neurology, in addition to EM.^8^,^9^

Residents appreciate having protected educational time and, compared to shorter daily formats, the blocked weekly didactic structure has demonstrated higher learner satisfaction, improved attendance, and fewer interruptions.^10^–^13^ While learners perceive improved learning with this format, studies have failed to demonstrate differences in objective outcomes such as scores on standardized tests or board examinations.^10^–^14^ However, given the perceived and logistical benefits, including improved attendance, which is essential to maintaining accreditation, combined with the nature of EM clinical schedules, the authors recommend the blocked weekly format.

The ACGME places certain requirements on programs regarding faculty participation in didactics. These include that each core faculty member must attend at least 20% of planned didactic experiences and that EM faculty members must present at least 50% of resident conferences.^2^ While there is limited data evaluating faculty conference attendance and objective learning outcomes, one study found that higher faculty conference attendance was associated with higher pass rates on EM oral boards for trainees.^15^ Additionally, residents perceive that faculty presence at conference facilitates learning.^16^,^17^ One approach to increase faculty presence at conference would be to offer incentives for attending conference.^18^ Providing continuing medical education credit for didactic conferences can also increase faculty attendance.^19^

Conference didactics are most often presented by faculty or residents.^8^,^9^,^16^,^15^,^20^ Some have advocated for residents to give didactic lectures to ease the burden on faculty time and sharpen resident public speaking skills.^21^ While residents perceive that faculty lectures greatly contribute to their educational experience,^11^,^16^ limited data has demonstrated that residents can learn from resident-given lectures, and that no difference in learning outcomes (e.g., test scores, board passage rates) were found between resident-given lectures vs faculty-given lectures.^15^,^20^,^22^ Additionally, it may be appropriate to incorporate other professionals (e.g., nurses, pharmacists) as lecturers depending on the topic. Smith et al found no difference between lecture evaluation scores for nurse-given lectures compared to faculty- and resident-given lectures.^23^

Given that the specialty of EM interfaces with many other disciplines, it may also be beneficial to incorporate multidisciplinary conferences with other medical professionals into the didactic curriculum to enable collaborative learning, coordinated patient care, and a better understanding of the roles of other professions.^24^–^26^ The ACGME recommends the inclusion of multidisciplinary conferences as part of the resident didactic experience.^2^ Limited research suggests that trainees value this type of experience^24^,^27^; however, robust objective data on learning outcomes are lacking.

Instruction should be tailored to the level of the learner.^28^,^29^ However, this may be especially challenging in program-wide didactic conferences in which the learners differ significantly in terms of stages of training and faculty are at varying career stages and experience. In recent years, we have seen the development of a national EM curriculum specific to the training level and the nearly universal presence of a dedicated intern orientation in residency programs.^30^,^31^ To date, there are no objective data evaluating training level-specific didactics on learning outcomes; however, faculty and residents have been shown to view this targeted instruction positively.^32^,^33^

Resident didactic instruction has traditionally been delivered via lectures despite calls for alternatives.^34^,^35^ Common criticisms of lectures include lack of engagement due to an emphasis on passive learning,^36^ overwhelming students’ ability to learn by providing too much information,^37^ and waning attention due to the duration of the session.^38^ Despite calls to minimize the use of lectures, data support their continued effectiveness as a teaching modality.^39^–^41^ The common criticisms can be overcome through intentional learner-centered instructional design.

Cognitive load theory states that there are three main components involved in the creation of long-term memories: intrinsic load; extraneous load; and germane load.^28^ While intrinsic load and germane load are generally fixed, extraneous load is highly modifiable and heavily influenced by the manner in which material is presented to learners.^28^ Since the amount of working memory is generally fixed for a given person at a set time, increases in extraneous load (i.e., presenting information in an overly complex manner) will detract from learning and retention.^28^ Therefore, instructors should focus on ensuring that talks are focused on delivery of information, while limiting unnecessary information or overly complex presentations of the information. Multimedia learning theory informs principles of slide design and is one effective method that can be used to increase the long-term retention of taught material^42^ (Table 3).

With regard to the duration of lectures given at conference, the notion that shorter may be better is based on data of learner attention spans.^45^ In a classic study of medical students, Stuart and Rutherford found that the attention span peaked at 10–15 minutes and fell steadily thereafter, with the authors recommending that lectures not exceed 25–30 minutes.^45^ In more recent years, we have seen the implementation of shorter lectures in EM both at the local and national level.^34^,^46^ Limited studies have compared shorter (8- to 30-minute) segments compared to the more traditional 50- to 60-minute lecture and found the learners typically prefer the shorter format^47^–^49^; however, few have looked at objective learning outcomes. One study by Bryner did evaluate knowledge acquisition and retention between 20-minute and 50-minute lectures and found no significant difference.^50^ More research is needed to determine the optimal length of didactic sessions with an emphasis on outcome-based evaluations.^51^ When it is not possible to reduce the duration of a lecture, incorporating pauses, interactive questioning, and intermittent summarization can re-engage learners and improve attention to the content.^52^

Handouts are an additional method to increase the effectiveness of lectures. While many lecturers will distribute copies of their presentations, a more effective technique is the concept of guided notes. Guided notes are a hierarchical outline of the presentation with key information intentionally left blank. Learners will “fill in the blanks” as the lecture progresses, thus increasing attention and discovering the relationships in the presented material. Additionally, the fact that the notes are mostly complete allows for effective note-taking and allows attention to be directed at the presenter instead of the notebook.^53^

While lectures can still be effective, active learning has been shown to positively impact objective learning outcomes, by incorporating other instructional techniques.^54^–^63^ Active learning is “any instructional method that engages students in the learning process”^64^ and can include techniques such as games, flipped classroom, audience response systems, case-based problems, and team-based activities.^6^

Real-time electronic broadcasts of lectures and video conferencing can be another good use of technology to support resident education.^66^ This has been demonstrated to be an effective educational model that is positively viewed by trainees and can improve access and attendance at didactic offerings for both residents and faculty.^67^–^69^ For training programs with multiple sites or that have struggled with maintaining the required attendance percentage for accreditation, this may be a valuable option to consider.

Our understanding of how learning occurs has evolved as cognitive scientists continue to refine effective methods for teaching and learning. Unfortunately, effective methods are often not incorporated into medical curricula. Educators should avoid using or encouraging the use of learner-initiated summarization, highlighting and underlining, mnemonics, imagery, and rereading as these techniques have not been shown to enhance learning.^70^ Effective techniques with a strong effect size include practice testing and distributed practice. Additionally, there is likely some benefit from the use of elaborative interrogation, self-explanation, and interleaving.^70^

Practice testing is the use of no- or low-stakes tests that can be completed independently by the learners. These can include recall via flashcards, practice problems, or traditional types of test questions.^70^ Teachers may choose to implement this technique using shared card decks or applications (apps), or web-based asynchronous question banks. Anonymous audience-response systems are popular and have also been shown to improve student learning in medical education.^71^,^72^ Distributed practice (also known as spaced repetition) refers to the spreading out of learning over time as opposed to massed practice or “cramming.”^70^ Implementation of this technique can be accomplished by content mapping that allows for repeated exposure to the concepts from prior didactics, the use of handouts or summarization materials between didactic sessions, or by using email to re-expose learners to the material.^73^

Elaborative interrogation involves the use of self-questioning to enhance learning. This would involve the learner seeking out the underlying rationale or etiology using questions such as “why does this occur?” Similarly, self-explanation involves directing learners to explain their logic during task completion.^70^ Educators can easily incorporate this technique through simple questioning exercises during their lectures. Interleaving is an education organizational technique in which multiple topics and themes are mixed and covered over time instead of having discrete blocks dedicated to single topics.^74^

The flipped classroom, also known as the reverse classroom,^75^ is an instructional design method in which independent learning, often via previously-viewed video lectures or pre-reading, is combined with face-to-face classroom activities.^76^ When studied, the flipped classroom appears to be effective^77^–^79^; however, caution should be exercised as recent systematic reviews have found high methodological diversity, inconsistent results, and risk of bias.^76^,^80^–^82^ Gamification is another active learning technique, which involves the utilization of games and competition to support learning.^83^ As a technique, gamification may support learning of skills,^84^ emergency department (ED) throughput,^83^ decision-making,^85^ and medical knowledge.^86^–^89^

Team-based learning (TBL) is an instructional method used with increased frequency in both undergraduate medical education and graduate medical education, which is often combined with the flipped classroom model.^90^–^93^ Prior to TBL, learners are expected to prepare and complete a pre-session test individually ahead of time. During the TBL sessions, learners then work in teams to solve a series of realistic, complex problems. Faculty serve as facilitators encouraging peer-learning, cooperation, and ensuring the discussion stays on track. This approach requires upfront training of faculty in discussion facilitation and learner buy-in to prepare for sessions.^91^,^94^

**BEST PRACTICE RECOMMENDATIONS**

1. Didactic lectures should be administered as blocked, weekly sessions (Level 2b; Grade B).
2. Encourage faculty attendance and participation in conference (Level 3b; Grade B).
3. Lecture can still be an effective method to present didactic content. When this technique is used, the lecturer should ensure that their presentation complies with cognitive load theory, multimedia learning theory, and active learning principles (Level 1a; Grade B).
4. Real-time video conferencing can be considered to improve access and attendance (Level 3b; Grade C).
5. Educators should incorporate the use of spaced repetition and no- or low-stakes testing into didactic instruction to increase long-term retention of content (Level 1a; Grade A).
6. Utilization of recorded lectures, flipped classroom, and gamification can supplement or replace the traditional lecture (Level 1a; Grade B).

### Conference Topics

After a thorough review of the literature, we found no prospective studies evaluating which specific topics should be included in the conference didactic curriculum. For this reason, the core content as described by the Model of the Clinical Practice of Emergency Medicine, or the “EM Model,”^95^ is most commonly used as the de facto foundation of the conference curriculum in most residencies. While this was designed using expert consensus data, it is heavily informed by those areas most relevant to the emergency physician. In fact, during the creation of the EM Model, hospital data from over 90 million ED visits were compared to its content and found to have 82% overlap, validating the content of the EM Model.^96^ The EM Model is further refined every three years to identify new areas to cover.^97^ As it is used to inform board certification examinations, it is important for residents to be familiar with all of the topics covered and is a critical initial reference for most conference planners.^98^ While there is no strong data to help prioritize specific subject matter during conference time, in-training examination coverage of various areas may help guide emphasis on high-yield topics.

While the EM Model may be used as a guide for resident education, conference didactics should be viewed only as one component of resident education with its unique strengths and weaknesses. As such, rather than focusing solely on “covering” all topics in the EM model, the priority of conference didactic design should be on maximizing the learning potential of this modality.^99^ Additionally, some topics can best be taught through other components of resident education including clinical experience, outside reading, simulation and use of Free Open Access Medical Education (FOAM).^4^

The ACGME Program Requirements for Graduate Medical Education (GME) in EM mandate specific conference content to be taught as part of didactics.^2^ These include five main components listed in Table 4.

Additionally, the ACGME requires a number of other specific themes to be included in residency training.^2^ We suggest incorporating the following into your conference topics to assure completion of these requirements.

#### Patient Safety and Quality Improvement

Residents should be educated in a culture of safety, including understanding safety goals, diagnostic error, response to adverse events, continuous quality improvement, and ultimate accountability of the physician for the care of the patient. This can also be combined with M&M conference sessions.^100^*Professionalism*

Residents must be aware of their professional responsibilities toward their patients and peers, as well as their relationship with the health system on a local and national level. Residents should also appreciate the necessity of their own need for ongoing education after residency and how to obtain and maintain board certification.

#### Well-Being

In recognition of the prevalence of depression, burnout, substance abuse, and suicidality among residents and medical students, the ACGME now mandates teaching on the identification and mitigation of these concerning issues. While there is no set curriculum provided or recommended by the ACGME itself, materials are available, such as the Educational Toolkit provided by the 2017 Resident Wellness Consensus Summit.^101^ This incorporates modules on second victim syndrome, mindfulness and mediation, and positive psychology.

#### Fatigue mitigation

All residents must be able to recognize limitations in their ability to care for patients due to sleep deprivation and fatigue; they should be made aware of options for fatigue management and transition of care to another provider, should the need arise.

Given the limited evidence-based data on curricular content of didactics further dedicated research on possible curricular content and the weighting of topics taught may be beneficial.

**BEST PRACTICE RECOMMENDATIONS**

1. Core content topics for conference should be derived from the conditions and skills described in the EM Model (Level 5, Grade D).
2. Curriculum presentations, morbidity and mortality sessions, research seminars, journal review, and administrative seminars should be included as part of the conference design (Level 5, Grade D).

## LIMITATIONS

There are several limitations to consider for this review. First, it is possible that some articles were not identified using our search strategy; however, an experienced medical librarian conducted the search with a broad search strategy using multiple databases. Additionally, we searched bibliographies of all included articles, contacted topic experts, and underwent pre-submission peer review by the entire CORD community.

Given the breadth of this topic, we were unable to address all aspects of conference planning and some components (e.g., simulation, journal club) were therefore not included in the current review. However, journal club was previously covered in a prior best practice manuscript,^3^ and other topics may be covered in future best practice recommendations. Moreover, some areas did not have EM-specific data available. When data specific to EM residency conference didactics was limited, relevant data from other specialties and fields was also incorporated.

## CONCLUSION

This article provides a summary of the best practice guidelines in developing resident didactic structure and content based on current literature and expert consensus. It offers a set of recommendations regarding the various didactic modalities, techniques to maximize the benefit from these sessions, and addresses effective incorporation of technology to improve participation. With regard to content, the authors recommend following the EM Model as a scaffold as well as incorporating other topics as specified by the ACGME such as research, professionalism, journal review, and wellness. More research is needed to better guide what content should be included in didactics and to what extent.

The authors hope that this review will serve as a blueprint for programs to optimize their conference curriculum, ensuring a more uniform, high-quality level of education for their residents. Ultimately, educational designers must create a curriculum in the context of their specific institution, balancing pedagogically robust didactic content and structure with the resources available to them including money, time, equipment, and space. It is our hope that this review can be used by educators to advocate for additional resources from their department or institution to better facilitate evidence-based education for residents.

## Figures and Tables

**Table 1:** Oxford Centre for Evidence-Based Medicine criteria.^7^

Level of Evidence	Definition
1a	Systematic review of homogenous RCTs
1b	Individual RCT
2a	Systematic review of homogenous cohort studies
2b	Individual cohort study or a low-quality RCT*
3a	Systematic review of homogenous case-control studies
3b	Individual case-control study**
4	Case series or low-quality cohort or case-control study***
5	Expert opinion

* <80% follow up;

** includes survey studies;

*** studies without clearly defined study groups.

*RCT*, randomized controlled trial.

**Table 2:** Oxford Centre for Evidence-Based Medicine grades of recommendation.^7^

Level of Evidence	Definition
A	Consistent level 1 studies
B	Consistent level 2 or 3 studies or extrapolations* from level 1 studies
C	Level 4 studies or extrapolations* from level 2 or 3 studies
D	Level 5 evidence or troublingly inconsistent or inconclusive studies of any level

* “Extrapolations” are where data is used in a situation that has potentially clinically important differences than the original study situation.

**Table 3:** Mayer’s 12 principles of multimedia learning.^43^,^44^

**Coherence Principle:** Avoid extraneous words, pictures, and sounds. They can detract from learning. **Signaling Principle:** Add cues to highlight the essential materials. **Redundancy Principle:** On-screen text can detract from learning. People learn better from graphics and narration alone as opposed to graphics, narration, and on-screen text. **Spatial Contiguity Principle:** Corresponding words and pictures should be presented near each other rather than far from each other on the screen. **Temporal Contiguity Principle:** Corresponding words and pictures should be presented simultaneously rather than successively. **Segmenting Principle:** Multimedia lessons should be presented in learner-controlled segments rather than as a continuous unit. **Pre-training Principle:** When students already know the names and behaviors of system components, they will learn more from the session. **Modality Principle:** Learning is more effective when words are presented as narration rather than on-screen text. **Multimedia Principle:** Learning is more effective when words are combined with pictures as opposed to include words alone. **Personalization Principle:** Information delivery is more effective when words are presented in a conversational style rather than formal style. **Voice Principle:** Learning is more effective when narration is spoken in a friendly human voice rather than a machine voice. **Image Principle:** Learning is not necessarily more effective when the speaker’s image is added to the screen

**Table 4:** Main components of conference didactics.

Curriculum presentations Quality improvement/morbidity and mortality Research seminars (including education on how to conduct and understand research in a clinical context) Journal review and evidence-based medicine concepts Administrative seminars (to include operations and administrative practices in emergency medicine)
